# Design and Implementation of Symmetric Legged Robot for Highly Dynamic Jumping and Impact Mitigation

**DOI:** 10.3390/s21206885

**Published:** 2021-10-17

**Authors:** Lei Wang, Fei Meng, Ru Kang, Ryuki Sato, Xuechao Chen, Zhangguo Yu, Aiguo Ming, Qiang Huang

**Affiliations:** 1 Intelligent Robotics Institute, School of Mechatronical Engineering, Beijing Institute of Technology, Beijing 100081, China; wanglei_bit@bit.edu.cn (L.W.); kangru@bit.edu.cn (R.K.); chenxuechao@bit.edu.cn (X.C.); yuzg@bit.edu.cn (Z.Y.); qhuang@bit.edu.cn (Q.H.); 2 Beijing Advanced Innovation Center for Intelligent Robots and Systems, Beijing Institute of Technology, Beijing 100081, China; ming@mce.uec.ac.jp; 3 Department of Mechanical Engineering and Intelligent Systems, The University of Electro-Communications, Tokyo 182-8585, Japan; sato_r@rm.mce.uec.ac.jp

**Keywords:** legged robot, leg topology, highly dynamic jumping, impact mitigation, nonlinear optimization

## Abstract

Aiming at highly dynamic locomotion and impact mitigation, this paper proposes the design and implementation of a symmetric legged robot. Based on the analysis of the three-leg topology in terms of force sensitivity, force production, and impact mitigation, the symmetric leg was designed and equipped with a high torque density actuator, which was assembled by a custom motor and two-stage planetary. Under the kinematic and dynamic constraints of the robot system, a nonlinear optimization for high jumping and impact mitigation is proposed with consideration of the peak impact force at landing. Finally, experiments revealed that the robot achieved a jump height of 1.8 m with a robust landing, and the height was equal to approximately three times the leg length.

## 1. Introduction

Legged robots have good potential for traversing difficult obstacles, and the execution of highly dynamic maneuvers—such as jumping and running—has attracted a lot of attention. Many state-of-the-art legged robots, such as Atlas [1], BHR [2], Bigdog [3], ANYmal [4], MIT Cheetah [5], Jueying [6], HyQ [7], SCalf [8], Minitaur [9], Stanford doggo [10], GOAT [11], and Salto [12], have achieved significant advancements. Many of the abovementioned legged robots can perform versatile and stable gait. However, the highly dynamic locomotion and corresponding physical interaction with the environment remain challenging.

Jumping is characterized by a large instantaneous ground reaction force (GRF) and short duration. Therefore, the impact mitigation is crucial for legged robots, because the components of the leg link, reduction device, and motor are fragile in terms of resisting a large impact force. The ground reaction force is determined by the leg topology and actuator, and there are countless design trade-offs and conflicts in the design goals. Hence, this study considered an actuator design and the synthesis of leg topologies to achieve the desired performance.

Leg design is often inspired by biological observation. Generally, the following leg topologies are used: prismatic [13], two joint series articulated [14], three joint series articulated (often redundantly) [15], parallel planar [16,17,18], symmetric parallel [10,19], and parallel spatial [11]. The prismatic leg is limited by its workspace, and the series articulated—particularly, the redundantly articulated leg—have an adequate motion range. However, one of the joints (typically, the knee joint) may be subjected to a heavy load or need high joint velocity when jumping. The parallel leg is advantageous for force production, but has poor efficiency when swinging back and forth. The parallel spatial leg is the best option for force production but the workspace is not sufficient, and moving interference may exist when combing is carried out for a biped or quadruped robot, which may limit the variety of athletic gait. Hence, this option should be considered with regard to the objective of this study.

The actuator system is a significant component in robotics and requires an elaborate design. Although hydraulic actuation can provide robustness against high-impact forces and satisfy the power requirements in terms of velocity, torque, and bandwidth, it is not ideal for application in everyday situations. Therefore, this study focused on electric actuation, such as direct-drive (DD) [19], quasi-direct-drive (QDD) [20], heavy gear motors (HGM) [21], and series-elastic-drive (SEA) [22,23]. Although DD has the advantage of high force transparency and control bandwidth, it also has a few disadvantages, including lower torque density and thermal Joule heating. The HGM is typically used in humanoids or high-load robots to enforce the torque ability, but has backdrivability and efficiency drawbacks. The QDD is a compromise for increasing the effective torque and maintaining the proprioceptive; therefore, its popularity is increasing. The SEA has been further developed to mitigate the negative gear train effects and torque sensing accuracy while maintaining the torque density. The SEA also offers improved efficiency and mechanical robustness by using an elastic spring. However, as a consequence, the control bandwidth is inherently reduced.

To improve the dynamic jumping ability and impact mitigation of a robot, various passive elastic elements are added [24,25]. However, it is difficult to synthesize the spring control with a robot model; this hinders fast movement, because the physical properties of elastic elements complicate the modeling and control. By improving the design, some robots [12] have achieved satisfactory vertical jumping ability and remarkable performance matching that of animals. However, these robots have small size, which may limit the scope of their application.

In motion planning and control, the physical, dynamic, and ground interactions are crucial to a robot’s ability. In some jumping robots, a simple heuristic virtual model control based on impedance control is used without considering the whole body dynamic model and impact force [11]. Additionally, various optimal trajectory methods based on numerical integration are under development [26,27], but these methods introduce a numerical error that is crucial for reliable tracking control when applied to actual robotic systems. Yanran et al. [20,28] proposed a mixed-integer convex optimization method, which avoids localized optima during dynamic motion planning by approximating nonlinear and nonconvex constraints as mixed-integer constraints. However, in this model, the impact force may be an average value that filters the peak impact force, and the entire body dynamic is ignored.

Hence, it is important to design a suitable leg topology and actuation scheme that can support each other to satisfy the design specifications for maximum dynamic legged mobility. The objective of this study was to achieve high jumping capability and impact mitigation. Hence, a robust robot with symmetric leg topology and a high torque density actuator was designed. Nonlinear dynamic trajectory planning with kinematic and dynamic constraints and ground physical interaction is proposed to investigate the dynamic motion. The main contributions of this study are as follows:

(1) High force production and impact mitigation are crucial for highly dynamic jumping. It was found that the symmetric leg structure is better for landing impact mitigation. In the jumping and landing process, the symmetric leg requires lower joint torque and resists a moderate joint torque impact.
(2) For jumping and landing locomotion, nonlinear dynamic optimization was carried out with consideration of the dynamic constraints and peak impact force to determine the jumping GRF and landing stiffness for high jumping and impact mitigation.

The rest of this paper is organized as follows. The leg topology and actuator design are introduced in Section 2. Section 3 presents the modeling, motion generation, and control approaches. The simulation and experimental results are described in Section 4. The conclusions and directions of future work are discussed in Section 5.

## 2. Leg Design

**Figure 1:**
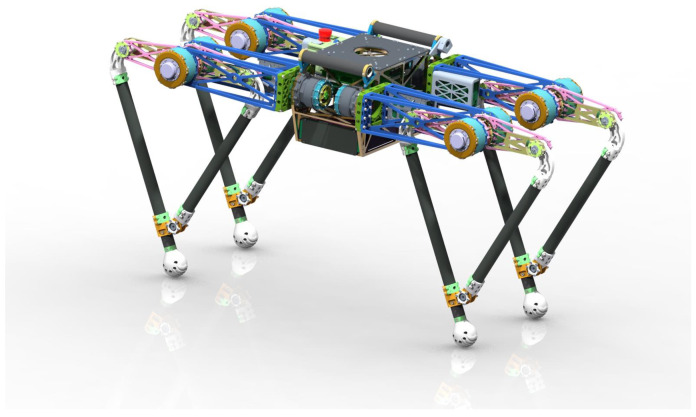
The BQR-2 robot.

The BQR, shown in Figure 1, is a quadrupedal robot with three actuated degrees of freedom (DOF) per leg. The flexion and extension of the leg is achieved by a symmetric structure. This study developed custom actuators consisting of brushless DC motors combined with two-stage integrated custom planetary gearboxes to provide high torque density at a small size and weight. The leg rod and body housing frame are made of carbon and connected by a high-strength aluminum alloy. The hip height is approximately 0.6 m. This study mainly focused on the leg geometry and actuators; therefore, the leg inertia was kept as low as possible to enable fast and efficient leg motion.

### 2.1. Leg Topology

**Figure 2:**
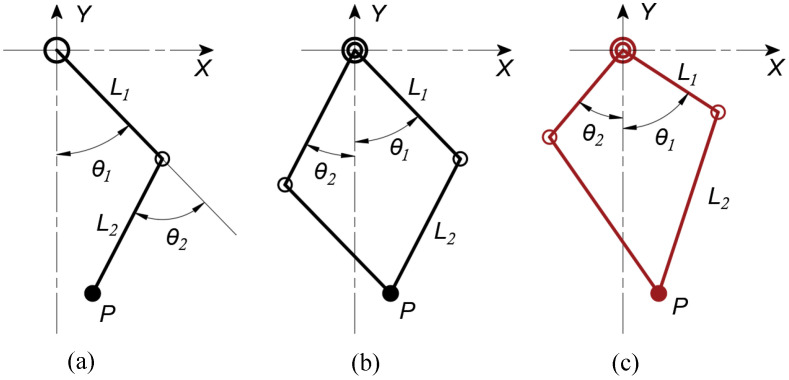
The three kinds of leg topology recruitment: (**a**) series articulated leg, (**b**) parallel planar leg, (**c**) symmetric leg.

Figure 2 shows the typical leg design topology. They are series leg (a serial chain of two revolute joints), parallel leg (a parallelogram four-bar), and symmetric leg (a symmetrical rhombus). $L_{1}$ and $L_{2}$ are the upper and lower linkage length, respectively, which are equal in the series and parallel leg topologies, and $L_{1}$ is half of $L_{2}$ in symmetric leg topology. This study mainly focused on three types of leg topology for proprioceptive force sensitivity, force production, and joint impact mitigation. Proprioceptive force sensitivity means that some forces change at the toe visible to the joint motor, according to Equation (1), where Δf is the force change unit. Hence, it is thought that proprioceptive sensing is strongly dependent on the leg topology determined by the Jacobian (Appendix A). As the transpose of the leg Jacobian relates the leg kinematic, more attention should be given to an ideal leg topology for accurate proprioceptive sensing.

(1) $$\tau =J^{T}\Delta f$$

**Figure 3:**
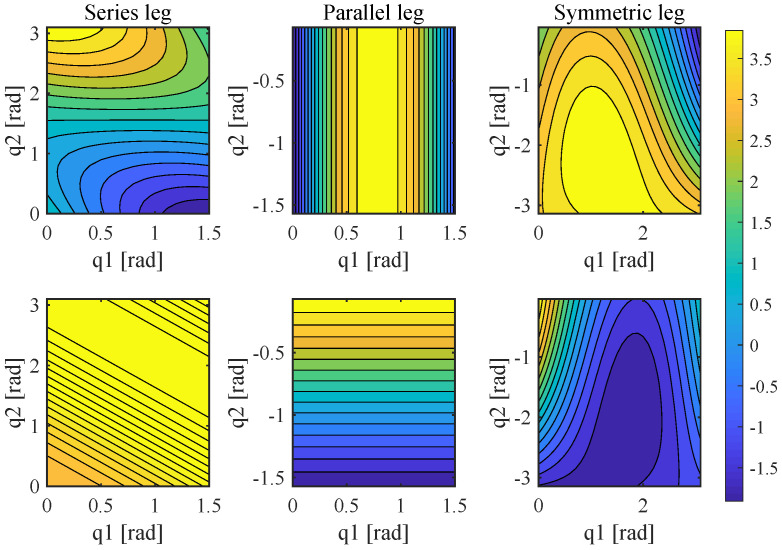
The proprioceptive sensing of three kinds of leg topology. The first and second line are joint 1 and joint 2 of the three types of leg, respectively; color bar represents the value (Nm) of torque of each joint.

Figure 3 shows the sensed proprioceptive force for every leg in the leg’s workspace. The coordinate on the plot corresponds to the range of motion of joint 1 and joint 2, respectively, and the contour plot’s color represents the value of torque of each joint actuator, corresponding to a foot force of 1 N. The force change of the end effector is mainly visible to one of the joints of a series. For a parallel leg, a particular workspace is better, and the symmetric leg mechanism generally has a better proprioceptive through most workspaces.

**Figure 4:**
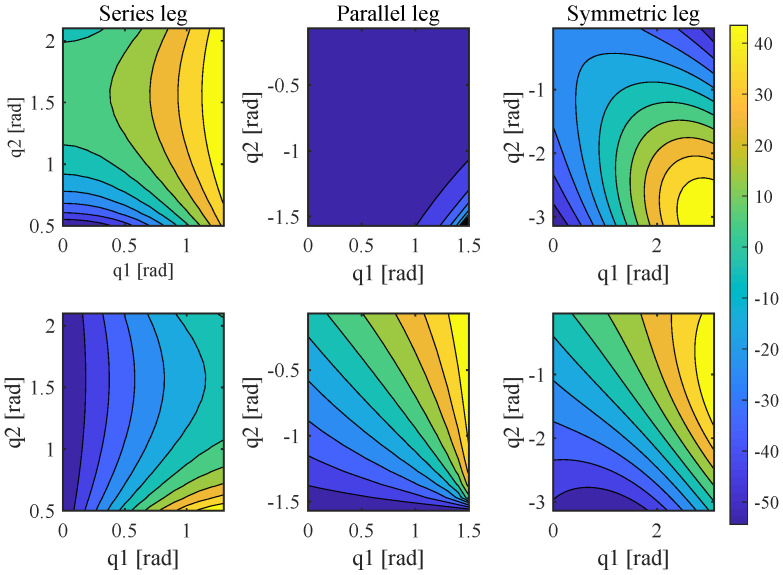
The force production of three kinds of leg topology. The first and second line are horizontal and vertical foot force of the three types of leg, respectively; color bar represents the force value (N).

Force production is a prominent metric of robot leg, a robot can jump higher and run faster with a large foot force, which is a prerequisite of agility. Figure 4 shows the force production calculated by Equation (2) for every leg in the leg’s workspace, and the contour plot’s color represents the value of force of each leg at the end effector corresponding to a joint torque of 10 Nm. The first line is the horizontal force and second line is the vertical force. From the Figure 4, it can be seen that the parallel and symmetric legs are better for force production and the series leg can produce a larger force in some particular workspace.

(2) $$f=J^{-T}\tau$$

**Figure 5:**
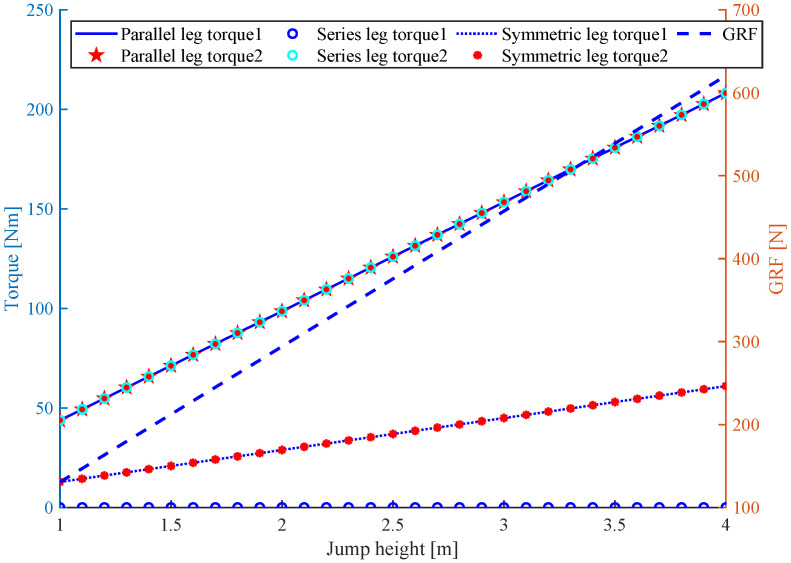
The max joint torque of three kinds of leg with increasing GRF, and the GRF corresponding to vertical jumping height.

Physical interactions with the environment play a crucial role in many legged robot applications. Legged locomotion involves repeated dynamic events such as instantaneous impact and continuous high-force interaction with uncertain terrain. The impact force at the touch down instance is crucial in robotics because a large ground impulse may break the gear teeth. Figure 5 shows the GRF and maximal joint torque at different jump heights. It is assumed that the robot jumps with a constant acceleration, and the acceleration will increase with the jump height, which results in the increase in GRF. Then, the joint torque can be calculated using (1). Obviously, the hip joint torque of a series leg is zero because a vertical jump is performed, the knee joint torque is equal to the double joint torque of the parallel leg, and the joint torque of the symmetric leg is the smallest. Hence, it is assumed that the symmetric leg joint can resist a moderate force at the same interaction force in vertical jumping and landing. Thus, the symmetric leg topology is robust and better for impact mitigation under the condition of vertical jumping.

In a word, compared with the series leg (such as MIT cheetah [5]), the parallel and symmetric leg topologies are better for fore production, and in the vertical jumping motion workspace, the symmetric leg requires lower joint torque, which means that a symmetric leg can resist a moderate joint torque impact when landing. Thus, the symmetric leg is better for high jumping and impact mitigation. The Minitaur [9] and Stanford doggo [10] are a direct and quasi-direct drive robot, but they are small size without enough load capability. In addition, compared with the symmetric leg, the series leg has a bigger limb workspace, which is better for ground clearance; the parallel and symmetric leg are not suitable for efficiency when the leg swings back or forward, because one motor is doing positive work while the other is doing negative work.

### 2.2. Actuator Design

**Figure 6:**
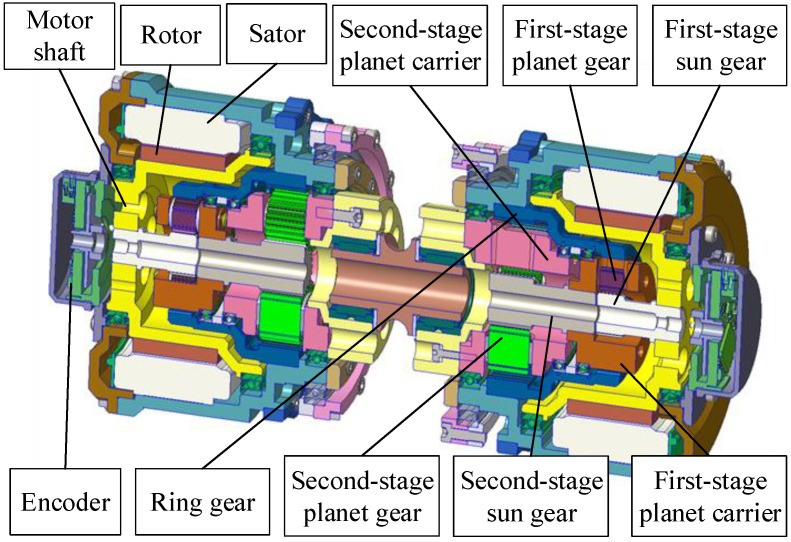
Actuator design. The actuator is designed with a custom motor and two-stage planetary gear for high torque density.

**Figure 7:**
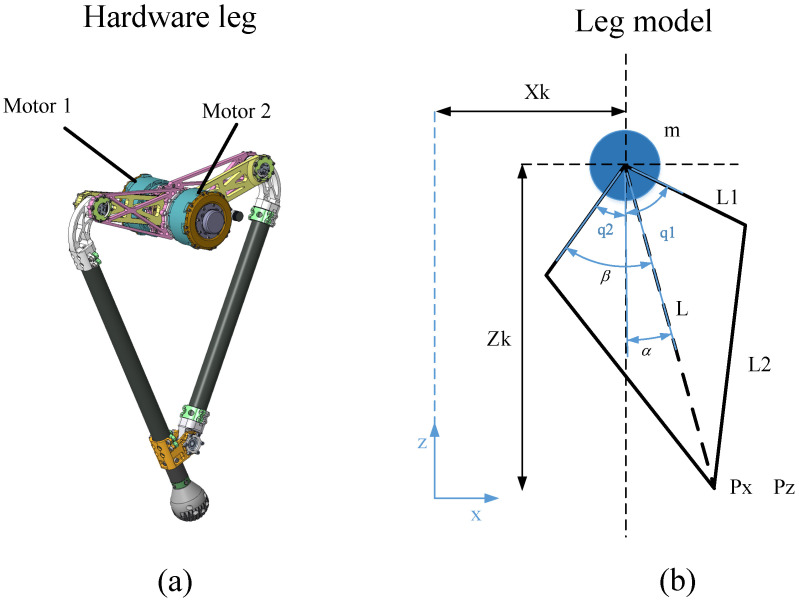
The leg model. (**a**) prototype of robot leg, (**b**) configuration parameters.

This study focused on the torque density [29] and size for an agile robot, which should have excellent acceleration. In order to improve the torque density, the torque should be large and the mass should be low. Compared with other agile legged robot with a common single-stage planetary gear (the reduction ratio is usually below 10), the two-stage planetary gear ratio is 17.4 in this paper to increase the output torque and thus, to increase the torque density. As shown in Figure 6, the first-stage sun gear coupled within the motor shaft, the first-stage planet carrier, and the second-stage planet carrier are all linked with the ring gear. It is clear that most of the two-stage planetary is inside the motor shaft to keep a compact configuration and to lessen the mass. The actuator parameters are listed in Table 1. It is expected that a small, large-reduction device combined with a counterpart motor will have an advantage over a large motor size [10]. However, this may reduce the transparency, and a trade-off between the abovementioned approaches should be made based on the specific application. Similarly, Cheetah 3’s actuators couple a single-stage planetary gear reduction, which is slightly higher compared with Cheetah 2, to improve the load-carrying ability and low-speed efficiency of the robot. Finally, a symmetric leg with a high-density actuator was designed, as shown in Figure 7a.

## 3. Nonlinear Optimization for High Jump and Impact Mitigation

### 3.1. Model and Analysis

Figure 7b shows the schematics of leg model, for which it is assumed that all mass is lumped at the base and the two motors are coaxial. Here, $P_{x}$, $P_{z}$ are the position of the foot in the body coordinates, and are expressed by Equation (3). $L_{1}$ and $L_{2}$ are the upper and lower leg length, *m* is the mass of body, $\beta =\frac{q_{1}-q_{2}}{2}$ is virtual angle between the vertical and virtual leg *L*, $\alpha =\frac{q_{1}+q_{2}}{2}$ is the half angle between two upper leg. $x_{k}$, $z_{k}$ is the position of center of mass (COM) in inertial coordinate.

(3) Px=sinα(L1cosβ+(L22−L12+L12cos2β)12)Pz=−cosα(L1cosβ+(L22−L12+L12cos2β)12)

The dynamics of leg model were formalized as a single point mass. Because the GRF is the only external force that determines the state of robot, we select it as the control input. The equation of COM dynamic motion is expressed by Equation (4).

(4) $$\left[\begin{array}{c} \ddot{x}\\ \ddot{z} \end{array}\right]=\frac{1}{m}\left[\begin{array}{c} F_{x}\\ F_{z} \end{array}\right]-\left[\begin{array}{c} 0\\ g \end{array}\right]$$

where *x* and *z* are the displacement of body; $F_{x}$ and $F_{z}$ are the GRF, which can be parameterized; and *g* is the gravitational acceleration.

In this study, $F_{x}$ and $F_{z}$ were parameterized with Mth-order Bézier polynomials defined at interval [0, T] [15,30]:

(5) $$B_{M}\left(s\right)=\sum_{i=0}^{M}\alpha_{i}B_{i,M}\left(s\right)$$

where $\alpha_{i}$ denotes the coefficients of the ith Bernstein basis $B_{i,M}$, whose derivative is expressed as follows:

(6) $$\frac{d}{ds}B_{i,M}\left(s\right)=\frac{M}{T}\left(B_{i-1,M-1}\left(s\right)-B_{i,M-1}\left(s\right)\right)$$

where *s* is the normalized time within the time interval [31]. This property can be used to obtain an analytical solution, and the start and end values only depend on the first and last coefficients, which are set to zero to ensure a smooth and physically feasible ground reaction force profile. In this study, a 5th-order Bezier polynomial is used to parameterize GRF profile, and its coefficients are

(7) $$\alpha_{f}=\left[0,\alpha_{f0},\alpha_{f1},\alpha_{f2},\alpha_{f3},\alpha_{f4},0\right]$$

As is known, by parameterizing the GRF as Bézier polynomials, the corresponding COM velocity and position also take the form of Bézier polynomials due to the property of Equation (6). Given the initial velocity ${\dot{x}}_{0}$ and ${\dot{z}}_{0}$, the velocity trajectory of COM in stance phase could be integrated analytically using Equations (4)–(6), which are also a Bezier polynomial with the coefficients $\alpha_{vx},\alpha_{vz}\in R^{M+2}$, respectively, which can be written as follows:

(8) $$\begin{array}{c} \left[\begin{array}{cccccc} \frac{-T_{st}}{6} & \frac{T_{st}}{6} & 0 & \cdots & 0 & 0\\ 0 & \frac{-T_{st}}{6} & \frac{T_{st}}{6} & \cdots & 0 & 0\\ \vdots & \vdots & \vdots & \ddots & \vdots & \vdots \\ 0 & 0 & 0 & \cdots & \frac{-T_{st}}{6} & \frac{T_{st}}{6}\\ 1 & 0 & 0 & \cdots & 0 & 0 \end{array}\right]\left[\begin{array}{c} \alpha_{vx0}\\ \alpha_{vx1}\\ \vdots \\ \alpha_{vx5}\\ \alpha_{vx6} \end{array}\right]\\ ={\left[\begin{array}{ccccc} \alpha_{fx0} & \alpha_{fx1} & \cdots & \alpha_{fx5} & m{\dot{x}}_{0} \end{array}\right]}^{T} \end{array}$$

(9) $$\begin{array}{c} \left[\begin{array}{cccccc} \frac{-T_{st}}{6} & \frac{T_{st}}{6} & 0 & \cdots & 0 & 0\\ 0 & \frac{-T_{st}}{6} & \frac{T_{st}}{6} & \cdots & 0 & 0\\ \vdots & \vdots & \vdots & \ddots & \vdots & \vdots \\ 0 & 0 & 0 & \cdots & \frac{-T_{st}}{6} & \frac{T_{st}}{6}\\ 1 & 0 & 0 & \cdots & 0 & 0 \end{array}\right]\left[\begin{array}{c} \alpha_{vz0}\\ \alpha_{vz1}\\ \vdots \\ \alpha_{vz5}\\ \alpha_{vz6} \end{array}\right]\\ ={\left[\begin{array}{ccccc} \alpha_{fz0}-mg & \alpha_{fz1}-mg & \cdots & \alpha_{fz5}-mg & m{\dot{z}}_{0} \end{array}\right]}^{T} \end{array}$$

Similarly, when given the initial CoM position $x_{0}$, $z_{0}$, the Bézier coefficients $\alpha_{px}$, $\alpha_{pz}$$\in R^{M+3}$ of the CoM trajectory can be obtained as follows:

(10) $$\Lambda \left(M+3,T_{st}\right)\alpha_{px}={\left[\begin{array}{cc} \alpha_{vx}/m & x_{0} \end{array}\right]}^{T}$$

(11) $$\Lambda \left(M+3,T_{st}\right)\alpha_{pz}={\left[\begin{array}{cc} \alpha_{vz}/m & z_{0} \end{array}\right]}^{T}$$

### 3.2. Nonlinear Optimization

The GRF is crucial for highly dynamic robot locomotion. Additionally, a large and suitable GRF is beneficial within the limitations of the actuator velocity, torque, and leg geometric constraints. However, an extremely large GRF may result in damage to the actuator gear or leg and body frame, particularly at a connection point. In this study, this was considered as an optimal control problem and solved using nonlinear optimization. The objective was to jump as high as possible and achieve a safe landing without any fracture. Hence, the goal function was formulated as expressed by Equation (12), which is a nonlinear and nonconvex problem:

(12) $$x_{opt}=\min\;-\frac{h_{max}}{F_{max}}$$

(13) $$\begin{array}{c} h_{max}=h_{0}+\frac{v_{z0}^{2}}{2g} \end{array}$$

(14) $$\begin{array}{c} F_{max}=max({\dot{z}}_{b}\sqrt{km}\sin\left(w_{n}t\right)+mg\left(1-\cos\left(w_{n}t\right)\right) \end{array}$$

where $h_{max}$ is the maximal jump height, which can be calculated using Equation (13); $h_{0}$ and $v_{z0}$ are the vertical height and velocity at taking off, which can be calculated by Equations (9) and (11), respectively. $F_{max}$ is the maximal impact force during landing, and can be obtained using Equation (14); ${\dot{z}}_{b}$ is the velocity before landing; *k* is leg stiffness interacting with ground; and $w_{n}=\sqrt{\frac{k}{m}}$ is the system natural frequency [32,33]. For simplicity, the velocity before landing is equal to the velocity at taking off. A larger taking-off velocity corresponds to a larger jumping height and a larger impact force, as shown by Equations (13) and (14); this is a conflicting requirement and can be solved by optimization. Hence, the optimal variables $x_{opt}$ can de defined as follows:

$$x_{opt}=\left[z_{0},\alpha_{fz(1-4)},T,k,q_{k(1-N)},{\dot{q}}_{{}_{k(1-N)}},{\ddot{q}}_{{}_{k(1-N)}}\right]$$

where $z_{0}$ is the initial vertical position of COM and *T* is the stance phase time. $\alpha_{fz(1-4)}$ are the Bezier polynomial coefficients of the vertical ground reaction force. $q_{k(1-N)}$, ${\dot{q}}_{{}_{k(1-N)}}$, and ${\ddot{q}}_{{}_{k(1-N)}}$ are the joint angle, angular velocity, and angular acceleration at each grid point, respectively; *N* denotes the sampled points. In the stance phase, it is assumed that the foot does not slip and some kinematics should be satisfied. The equality constraints can be expressed as follows:

(15) $$\left[\begin{array}{c} x_{k}\\ z_{k} \end{array}\right]+\left[\begin{array}{c} P_{x}\\ P_{y} \end{array}\right]=0$$

(16) $$J\left(q_{k}\right){\dot{q}}_{k}+\left[\begin{array}{c} {\dot{x}}_{k}\\ {\dot{z}}_{k} \end{array}\right]=0$$

where $q_{k}=\left[q_{k}^{1},q_{k}^{2}\right]$ and ${\dot{q}}_{k}=\left[{\dot{q}}_{k}^{1},{\dot{q}}_{k}^{2}\right]$. $P_{x}$, $P_{y}$, $x_{k}$, $z_{k}$, ${\dot{x}}_{k}$, and ${\dot{z}}_{k}$ can be calculated from Section 3.1. In the vertical jumping process, there is no horizontal movement; hence, $x_{k}=0$ and ${\dot{x}}_{k}=0$. The inequality constraints on joint angle, velocity, torque, geometry, and contact can be formulated as expressed by Equations (17), (19), and (20).

(17) $$\begin{array}{c} q_{lb}\leq q_{k}\leq q_{ub}\;\\ {\dot{q}}_{lb}\leq {\dot{q}}_{k}\leq {\dot{q}}_{ub}\;(k=1,2,\cdots ,N)\;\\ \tau_{lb}\leq \tau_{k}\leq \tau_{ub} \end{array}$$

where $q_{lb},q_{ub},{\dot{q}}_{lb},{\dot{q}}_{ub},\tau_{lb},\tau_{ub}$ are the lower and upper bounds of joint angle, angular velocity, and torque, respectively. Additionally, $\tau_{k}$ could be calculated by Equation (18). So, the torque constraint is a nonlinear inequality constraint. In highly dynamic jumping motion, the leg dynamic constraint should be satisfied, where ${\ddot{q}}_{k}=\left[{\ddot{q}}_{k}^{1},{\ddot{q}}_{k}^{2}\right]$, and the M,C,G, and $J_{k}$ are the mass matrix, Coriolis force, gravitation force, and contact Jacobian, respectively. Considering the range of motion and actual jumping, some margin should be secured as Equation (19). The friction cone limit on the foot was adopted with a static friction as expressed by Equation (20).

(18) $$\begin{array}{c} M{\ddot{q}}_{k}+C{\dot{q}}_{k}+G=\tau_{k}+J_{k}^{T}F_{k} \end{array}$$

(19) $$\begin{array}{c} \begin{array}{c} z_{0}\geq L2-L1\;\\ h_{0}\leq 0.85\left(L2+L1\right) \end{array} \end{array}$$

(20) $$\begin{array}{c} F_{x}\leq \mu F_{z} \end{array}$$

Similarly, optimization can be carried out for horizontal jumping. Then, the initial position of COM $x_{0}$, and horizontal GRF Bezier coefficients $\alpha_{fx(1-4)}$ should be added to the optimal variables. Additionally, we should change the cost function as the horizontal jumping distance:

(21) $$l_{\max}=l_{0}+v_{x0}\frac{v_{z0}}{g}$$

where $l_{0}$ and $v_{x0}$ are the horizontal distance and velocity at taking off, and can be calculated by Equations (8) and (10); then, we can obtain the GRF curves in the vertical and horizontal directions.

## 4. Simulation and Experiments

### 4.1. Simulation

**Figure 8:**
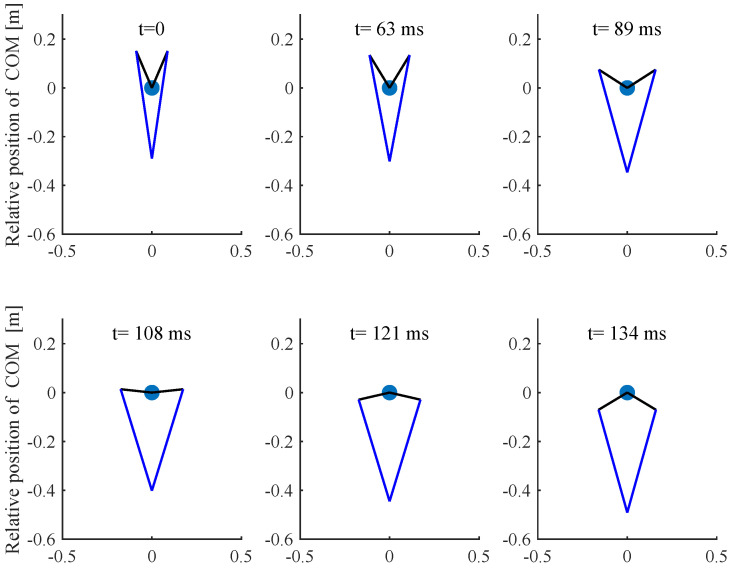
The simulation of vertical jump.

**Figure 9:**
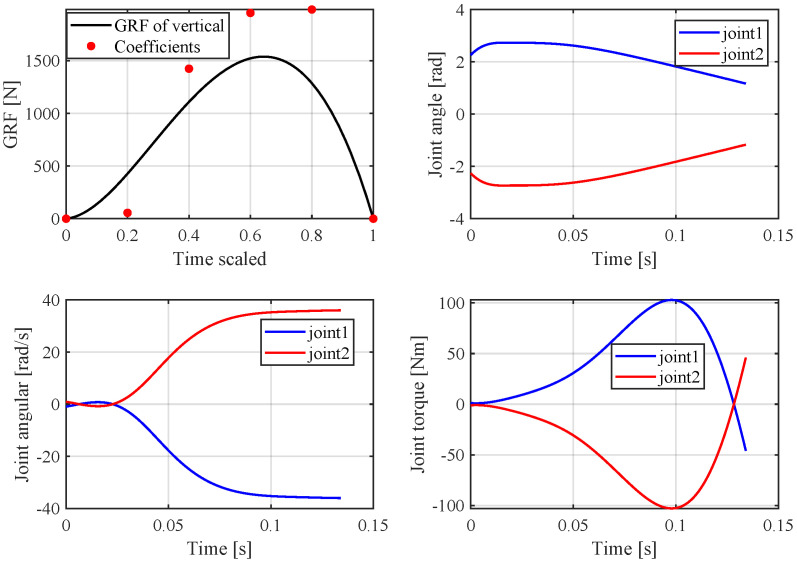
The results of vertical jump.

**Figure 10:**
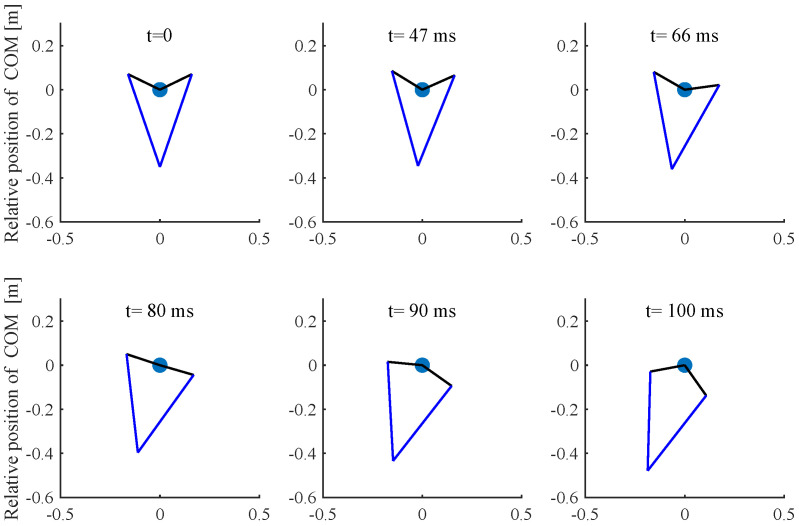
The simulation of horizontal jump.

**Figure 11:**
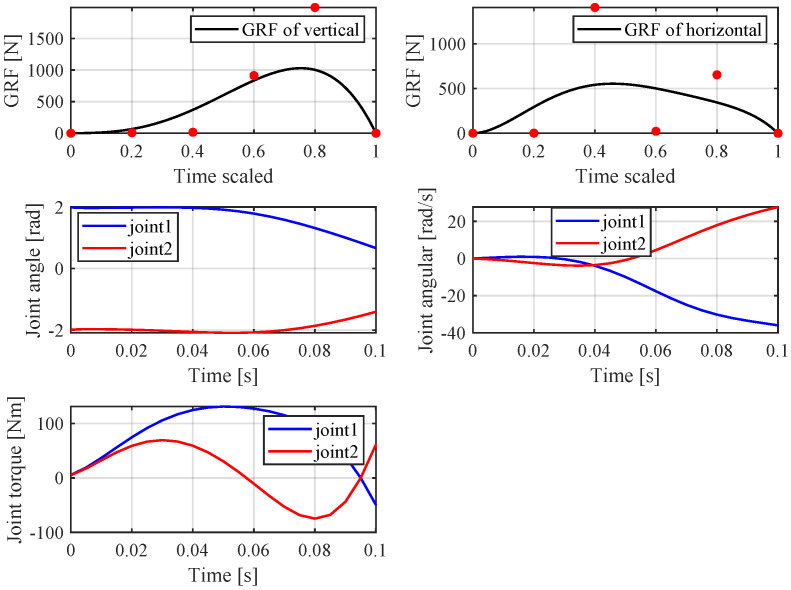
The simulation results of horizontal jump.

Based on previous analysis, a simulation was performed in MATLAB, and the *fmnicon* function was used to search for a optimal solution. In this paper, the cost function and dynamic constraints are nonlinear and nonconvex due to the trigonometrical terms and this often causes the optimization problem to converge to local optima. Thus, in order to obtain the global minimum solution, a local optimization was performed with multiple initial values using the MATLAB *multistar* function with multiple initial value; in this paper, the initial value number is 10,000. The Parallel Computing Toolbox was used to improve the simulation speed. In this simulation, the robot mass is 8 kg and other parameters are listed in Table 1. In the vertical jumping experiment, the following simulation results were obtained: $z_{0}$ = 0.29 m, *T* = 134 ms, *k* = 2800, $\alpha_{fz}$ = [0, 55.7, 1423.5, 1952.8, 1984.9, 0]; the simulation jumping height is 2 m. Figure 8 shows the vertical jump process, and Figure 9 shows the optimal results of GRF, joint angle, angular velocity and calculated joint torque. The red point indicates the Bezier polynomial coefficients of the vertical ground reaction force. Then, the entire curve can be obtained by Equation (5). The joint angle and angular velocity are the sequence of optimal variables $q_{k(1-N)}$ and ${\dot{q}}_{{}_{k(1-N)}}$, respectively. Finally, the joint torque can be calculated by Equation (18). With regard to horizontal jumping, the following simulation results were obtained: $z_{0}$ = 0.35 m, *T* = 100 ms, and $\alpha_{fz}=\left[0,8.9,15.3,912,1990,0\right]$, $\alpha_{fx}=\left[0,1.7,1406,21.7,65.3,0\right]$. Figure 10 and Figure 11 show the results of horizontal jump. From the optimal results, we can see that the maximum value of GRF, joint angle, angular velocity, and joint torque are all within the available range listed in Table 1 and almost reach the max value. In vertical jumping, there is a little squatting down in the initial stage; in horizontal jumping, the horizontal force plays a main role at the beginning and is then governed by the vertical force; the simulation force profiles are similar to human jumping data records [34,35].

### 4.2. Experiments and Discussion

**Figure 12:**
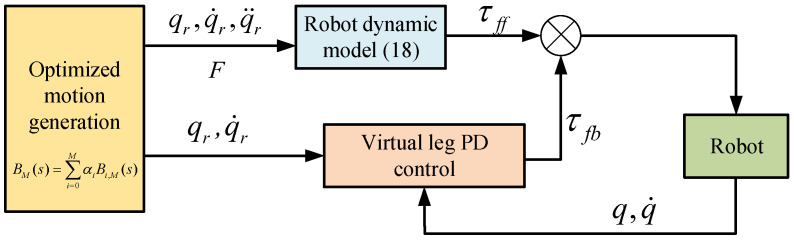
The diagram of robot controller.

The vertical jumping experiments were conducted using one of the symmetric legs, which was mounted onto a custom vertical rail. The experimental condition is the same as the simulation listed in Table 1. The drivers were installed on a board at the top of the robot; the power supply and computer were located off-board, and a 12-bit magnetic encoder was used to record motor angle. The control bandwidth was 1000 Hz and the force sensor was sampled using a computer program. The control block diagram is shown in Figure 12. The torque obtained by Equation (18) was considered as the feedforward torque. To ensure safety, the torque produced by the PD control of the virtual leg from the joint encoder was considered as the feedback torque, and the torque was governed by (22).

(22) $$\tau_{k}=\tau_{k}{,}_{ff}+J^{T}(K_{P}\left(l_{k}{,}_{r}-l\right)+K_{d}\left({\dot{l}}_{k}{,}_{r}-\dot{l}\right)$$

where the $K_{p}$ and $K_{d}$ are virtual leg stiffness and damping, respectively; $l_{k}{,}_{r}$, ${\dot{l}}_{k}{,}_{r}$, *l*, and $\dot{l}$ are the reference leg length, its velocity, actual leg length, its velocity, respectively.

**Figure 13:**
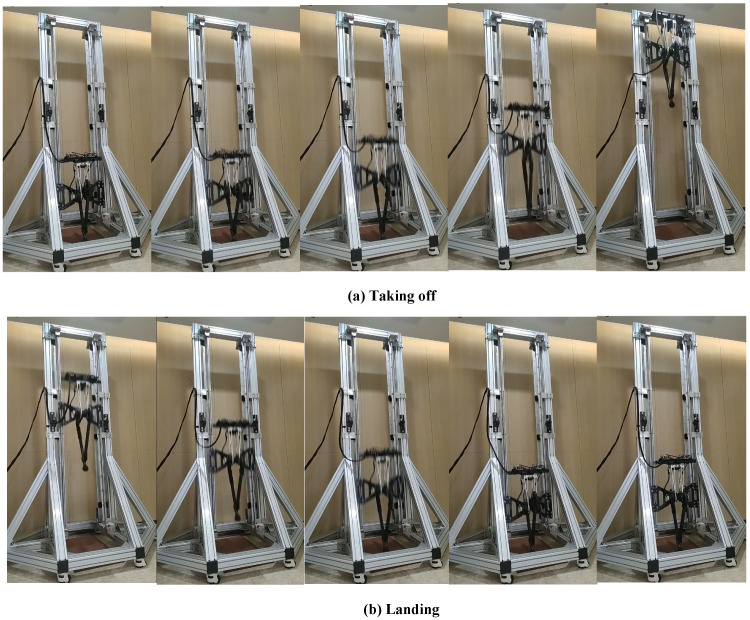
Snapshots of vertical jumping experiments.

**Figure 14:**
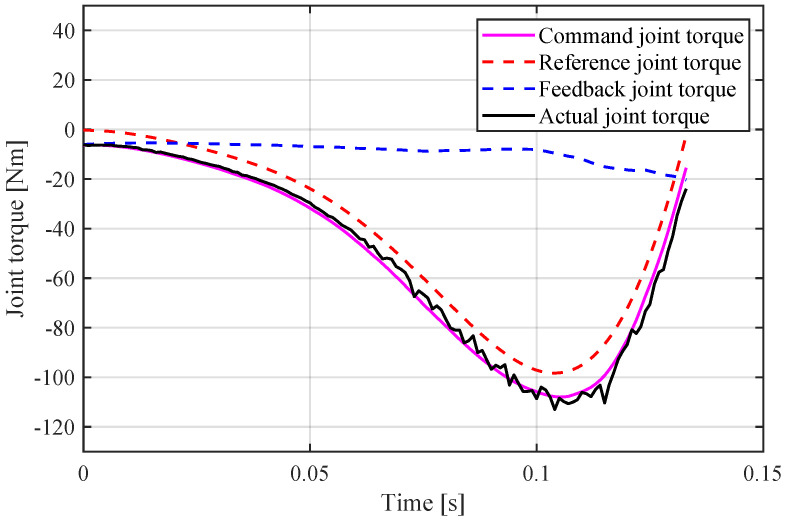
Joint torque of experiment.

**Figure 15:**
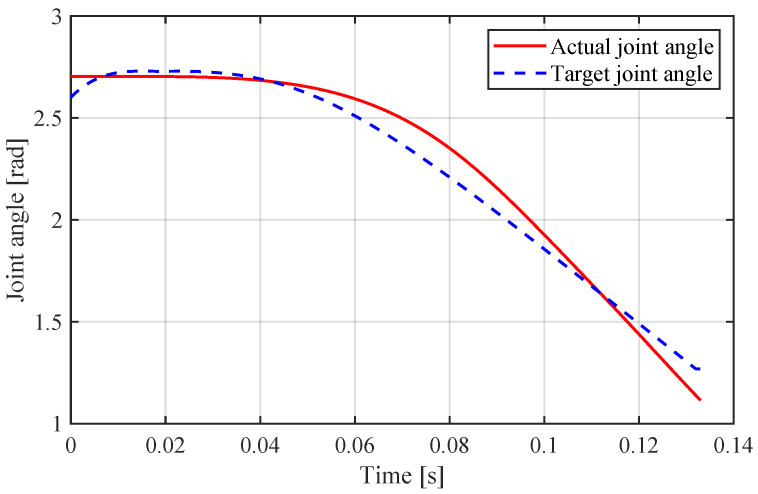
Joint angle of experiment.

**Figure 16:**
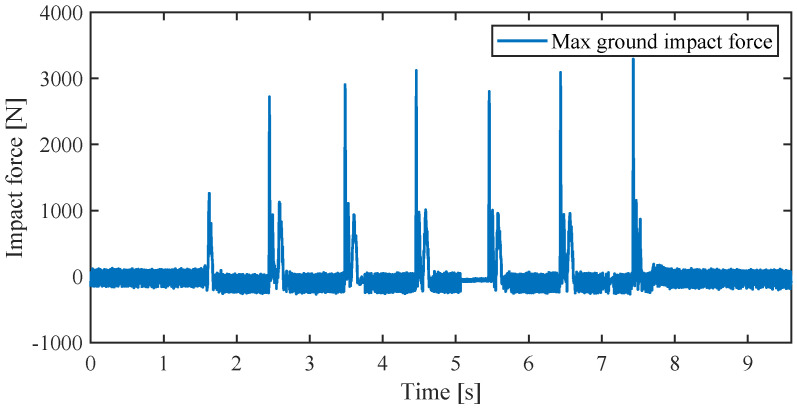
The experiment vertical jumping impact force.

Figure 13 shows the sequential snapshots. The robot jumped from a crouch condition and maintained a constant state after taking off. We took the video of jumping motion with a camera, and used the tracking data of the video and the reference mark on the auxiliary frame to measure the jumping height. Additionally, the reached height of the COM was approximately 1.8 m, which is equal to three times the leg length. The experimental height is lower than simulation, which may be caused by the friction of rail and the extra mass of auxiliary device. Figure 14 and Figure 15 show the data measured and planning. The actual torque was calculated using Equation (23).

(23) $$\tau_{\mathrm{actual}}=K_{T}I_{\mathrm{actual}}$$

where $K_{T}$ is the current constant, which was calibrated through a large number of tests to ensure high fidelity between the motor current and the joint torque. A certain error exists between the experimental data and the planning data, and may have been caused by the friction of the rail, which was ignored in this study, and the viscous friction when the angular velocity was high. The reason for this is the difficulty in calibrating the current constant when the motor speed is too high. So, the viscous friction caused by the high angular velocity is ignored. The small error of angle at the early stage of stance phase might be caused by the mass of auxiliary device. In the landing process, the virtual leg stiffness was set by the optimal value. Figure 16 shows the impact force after every landing. The maximum impact force was approximately 3000 N, which is approximately two times equal to the maximum end effector force and within the gear backdrive impact maximum torque.

## 5. Conclusions

This study developed a highly dynamic legged robot with an enhanced capability of high jumping and impact mitigation. A leg topology and nonlinear problem are presented in this paper. First, a detailed comparison between the series, parallel, and symmetric legs was carried out in terms of force sensitivity, force production, and impact mitigation. It was found that the symmetric leg structure is better for jumping and landing impact mitigation. Then, a symmetric leg with a custom motor and two-stage planetary gear was designed and implemented. Next, the nonlinear optimization for high jumping and landing impact mitigation was formulated. Finally, this study experimentally validated that the robot achieved a jumping height equal to three times the robot leg length and a soft landing. The robot system was robust after many jumps. In general, the main contributions of this study are that the symmetric leg structure is better for landing impact mitigation, and a jumping height (1.8 m) three times that of the robot leg length can be achieved by the nonlinear optimization for high jumping and landing impact mitigation. In future work, a horizontal experiment will be conducted, and a more detailed model, such as an actuator with a dynamic character, will be considered and will include the motor inertial and gear friction. Future work will also consider more than one leg of the quadruped robot and the model predictive control will be considered for more complex locomotion, using other optimization packages for efficient solving.

## Figures and Tables

**Table 1:** Actuator Parameter.

Parameter	Value	Units
Gear Ratio	17.4	
Motor Max Torque	7.8	Nm
Gear Backdrive Impact Max Torque	380	Nm
Max Joint Speed	36	Rad/s
Max End Effector force	1600	N

## Appendix A

(A1) $$J_{se}=\left[\begin{array}{cc} L_{2}\cos\left(\theta_{1}+\theta_{2}\right)+L_{1}\cos\theta_{1} & L_{2}\cos\left(\theta_{1}+\theta_{2}\right)\\ L_{2}\sin\left(\theta_{1}+\theta_{2}\right)+L_{1}\sin\theta_{1} & L_{2}\sin\left(\theta_{1}+\theta_{2}\right) \end{array}\right]$$

(A2) $$J_{pa}=\left[\begin{array}{cc} L_{1}\cos\theta_{1} & L_{2}\cos\theta_{2}\\ L_{1}\sin\theta_{1} & L_{2}\sin\theta 2 \end{array}\right]$$

(A3) $$J_{sy}=\left[\begin{array}{cc} {J_{sy}}^{11} & {J_{sy}}^{12}\\ {J_{sy}}^{21} & {J_{sy}}^{22} \end{array}\right]$$

where $J_{pa},J_{pa},J_{sy}$ are the Jacobians of the series, parallel, and symmetric legs, respectively.

(A4) $${J_{sy}}^{11}=\frac{\sqrt{2}(\left(2{L_{2}}^{2}-{L_{1}}^{2}\right)\cos\left(\frac{\theta_{1}+\theta_{2}}{2}\right)+{L_{1}}^{2}\cos\left(\frac{3\theta_{1}-\theta_{2}}{2}\right)}{4\sqrt{{L_{1}}^{2}\cos\left(\theta_{1}-\theta_{2}\right)+2{L_{2}}^{2}-{L_{1}}^{2}}}+\frac{\sqrt{2}L_{1}\cos\theta_{1}}{4}$$

(A5) $${J_{sy}}^{12}=\frac{\sqrt{2}(\left(2{L_{2}}^{2}-{L_{1}}^{2}\right)\cos\left(\frac{\theta_{1}+\theta_{2}}{2}\right)+{L_{1}}^{2}\cos\left(\frac{3\theta_{1}-\theta_{2}}{2}\right)}{4\sqrt{{L_{1}}^{2}\cos\left(\theta_{1}-\theta_{2}\right)+2{L_{2}}^{2}-{L_{1}}^{2}}}+\frac{\sqrt{2}L_{1}\cos\theta_{2}}{4}$$

(A6) $${J_{sy}}^{21}=\frac{\sqrt{2}(\left(2{L_{2}}^{2}-{L_{1}}^{2}\right)\sin\left(\frac{\theta_{1}+\theta_{2}}{2}\right)+{L_{1}}^{2}\sin\left(\frac{\theta_{1}-3\theta_{2}}{2}\right)}{4\sqrt{{L_{1}}^{2}\cos\left(\theta_{1}-\theta_{2}\right)+2{L_{2}}^{2}-{L_{1}}^{2}}}+\frac{\sqrt{2}L_{1}\sin\theta_{1}}{4}$$

(A7) $${J_{sy}}^{22}=\frac{\sqrt{2}(\left(2{L_{2}}^{2}-{L_{1}}^{2}\right)\sin\left(\frac{\theta_{1}+\theta_{2}}{2}\right)+{L_{1}}^{2}\sin\left(\frac{\theta_{1}-3\theta_{2}}{2}\right)}{4\sqrt{{L_{1}}^{2}\cos\left(\theta_{1}-\theta_{2}\right)+2{L_{2}}^{2}-{L_{1}}^{2}}}+\frac{\sqrt{2}L_{1}\sin\theta_{2}}{4}$$
